# Proposal to recognise the tribes Adinobotryeae and Glycyrrhizeae (Leguminosae subfamily Papilionoideae) based on chloroplast phylogenomic evidence

**DOI:** 10.3897/phytokeys.181.71259

**Published:** 2021-09-02

**Authors:** Lei Duan, Li-Na Han, Yotsawate Sirichamorn, Jun Wen, James A. Compton, Shuang-Wen Deng, Emine Arslan, Kuddisi Ertuğrul, Brian Schrire, Hong-Feng Chen

**Affiliations:** 1 Key Laboratory of Plant Resources Conservation and Sustainable Utilization, South China Botanical Garden, Chinese Academy of Sciences, Guangzhou 510650, China South China Botanical Garden, Chinese Academy of Sciences Guangzhou China; 2 College of Forestry and Landscape Architecture, South China Agricultural University, Guangzhou 510642, China South China Agricultural University Guangzhou China; 3 Silpakorn University, Department of Biology, Faculty of Science, Sanam Chandra Palace Campus, Nakhon Pathom 73000, Thailand Silpakorn University Nakhon Pathom Thailand; 4 Department of Botany, National Museum of Natural History, MRC 166, Smithsonian Institution, Washington D.C. 20013-7012, USA Smithsonian Institution Washington United States of America; 5 Spilsbury Farm, Tisbury, SP3 6RU, UK Spilsbury Farm Tisbury United Kingdom; 6 Department of Biology, Faculty of Science, Selçuk University, Konya 42031, Turkey Selçuk University Konya Turkey; 7 Accelerated Taxonomy Department, Royal Botanic Gardens, Kew, Richmond, Surrey, TW9 3AE, UK Royal Botanic Gardens Richmond United Kingdom

**Keywords:** *Adinobotrys*, Fabaceae, genome skimming, *Glycyrrhiza*, IRLC legumes, the GAW clade, Wisterieae

## Abstract

Within the legume family, the taxonomic status of subtribe Glycyrrhizinae of tribe Galegeae and of the genus *Adinobotrys* has been re-assessed. Based on genome skimming data, we conducted phylogenomic analyses of the inverted repeat-lacking clade within subfamily Papilionoideae. The results support the sister relationship between Glycyrrhizeae and *Adinobotrys*. Glycyrrhizeae is resurrected based on *Glycyrrhiza* and *Glycyrrhizopsis*, and a new tribe, Adinobotryeae, is proposed to accommodate *Adinobotrys*.

## Introduction

Within subfamily Papilionoideae of Leguminosae, a series of molecular phylogenetic studies have supported a monophyletic group that lost one copy of the 25-kilobase inverted repeat (IR) in the plastid genome, known as the “IR-lacking clade” or IRLC (Lavin et al. 1990; Sanderson and Liston 1995; Sanderson and Wojciechowski 1996; Doyle et al. 2000; Wojciechowski 2003). The IRLC harbours many economically important plants, for example, alfalfa, beans, clovers, lentils, peas, vetches, chickpeas and liquorice (Lewis et al. 2005). Recently, phylogeneticists have revealed an early branching clade, the Glycyrrhizeae-*Adinobotrys*-Wisterieae clade [i.e. the GAW clade named by Duan et al. (2021)], as sister to the remaining IRLC taxa (LPWG 2013, 2017; Duan et al. 2020, 2021; Xia et al. 2021).

**Figure 1.**
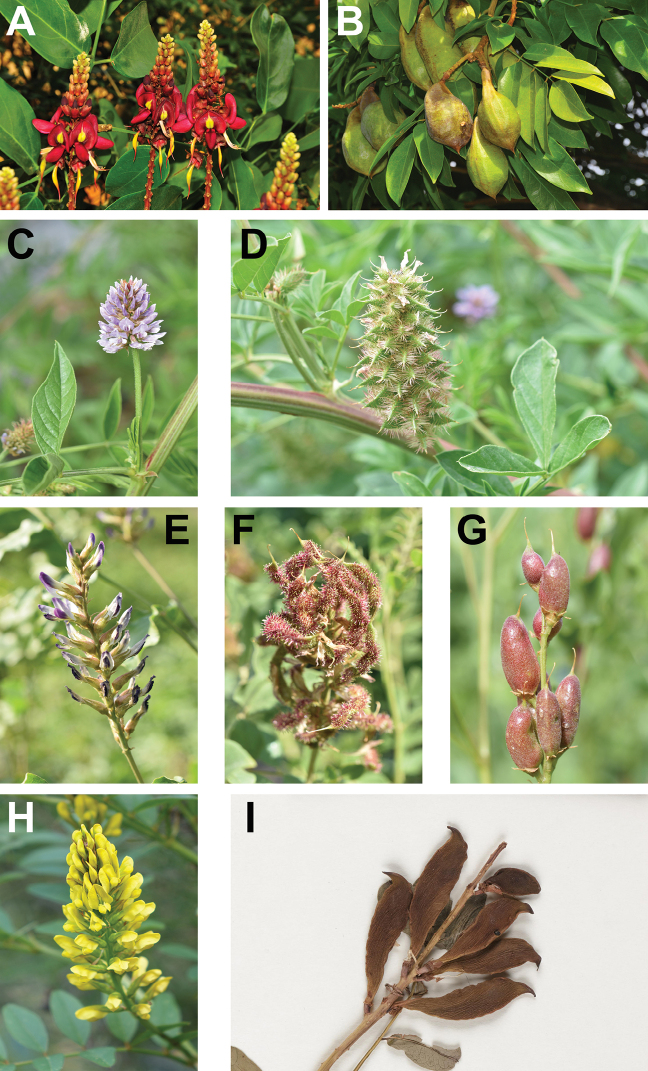
Representative plants of Adinobotryeae and Glycyrrhizeae. Inflorescences (**A**) and fruits (**B**) of *Adinobotrysatropurpureus*; inflorescence (**C**) and infructescence (**D**) of *Glycyrrhizapallidiflora*; inflorescence (**E**) and infructescence (**F**) of *Glycyrrhizauralensis*; fruits (**G**) of *Glycyrrhizainflata*; inflorescence (**H**) and part of dried infructescence [**I**; photographed on herbarium specimen: A. Eustace 31 (E!)] of *Glycyrrhizopsisflavescens*.

Within the GAW clade, the temperate herbaceous genera *Glycyrrhiza* L., with *Meristotropis* Fisch. & C.A.Mey. being treated within *Glycyrrhiza*, and *Glycyrrhizopsis* Boiss. are sister taxa (Duan et al. 2020, 2021; also see Fig. 1), Tribe Glycyrrhizeae was proposed by Rydberg (1917) to accommodate *Glycyrrhiza*, which was degraded as subtribe Glycyrrhizinae of tribe Galegeae by Rydberg (1923) and was then slightly emended by Polhill (1981) with *Glycyrrhizopsis* species treated as a synonym of *Glycyrrhiza*. Although Glycyrhizeae was not widely accepted by taxonomists, the taxonomic position of Glycyrrhizinae is also questionable. Galegeae is a polyphyletic and morphologically diverse tribe (Sanderson and Liston 1995; Lock and Schrire 2005; Duan et al. 2015), and our prior phylogenetic studies have shown that Glycyrrhizinae is only distantly related to core Galegeae (Duan et al. 2015, 2021). In consequence, the taxonomic rank of Glycyrrhizeae/Glycyrrhizinae needs to be reconsidered.

Apart from Glycyrrhizinae, the remaining taxa of the GAW clade included some woody genera, formerly placed in tribe Millettieae s.l., which were subsequently assigned into two clades: the liana (rarely climbing shrubs) tribe Wisterieae and the tree genus *Adinobotrys* Dunn (Compton et al. 2019; Duan et al. 2021; also see Fig. 1). Previous phylogenetic results excluded *Adinobotrys* from Wisterieae or Millettieae (Hu et al. 2000; Duan et al. 2021), but further taxonomic study is required to re-assess the placement of this genus.

In the present study, we used the genome skimming method (Straub et al. 2012; Zhang et al. 2015) to obtain 75 chloroplast (cp) coding sequences (CDSs), and constructed a phylogenomic framework of the GAW clade to test the taxonomic position and status of genus *Adinobotrys* and tribe Glycyrrhizeae. Our study provides a taxonomic foundation for future evolutionary, systematic and biogeographical studies of the IRLC legumes.

## Material and methods

### Taxon sampling

Sampling for molecular phylogenetic analyses included the genus *Adinobotrys* (one species sampled), both of the genera of tribe Glycyrrhizeae (eight species sampled) and 14 of the 15 genera (*Serawaia* J.Compton & Schrire not included) within tribe Wisterieae (26 spp. sampled) (Compton et al. 2019; Compton and Schrire 2020; Duan et al. 2020, 2021). Following Wojciechowski et al. (2004) and Duan et al. (2021), ten other genera of the IRLC (10 spp.) and three genera of Robinioids were selected as outgroups. Most samples for the study were obtained from fresh field collections or from preserved herbarium specimens (see Table S1 in the supplementary file for details), except that DNA samples of *Austrocalleryamegasperma* (F.Muell.) J.Compton & Schrire, *Whitfordiodendronnieuwenhuisii* (J.J.Sm.) Dunn, *Wisteriabrachybotrys* Siebold & Zucc. and *Wisteriafloribunda* (Willd.) DC. were accessed from the DNA and Tissue Bank, Royal Botanic Gardens, Kew (https://dnabank.science.kew.org), and the chloroplast genome of *Lotusjaponicus* (Regel) K.Larsen was downloaded from GenBank.

### DNA Extraction, Genome Assembly, Annotation and Alignment

We extracted the total genomic DNA following a modified CTAB protocol (Doyle and Doyle 1987). Yield and integrity (size distribution) of genomic DNA extracts were quantified by fluorometric quantification on a Qubit (Invitrogen, Carlsbad, California, USA) using a dsDNA HS kit, as well as by visual assessment on 1% agarose gels. Subsequently, we used all samples to build blunt-end DNA libraries using the NEBNext Ultra II DNA library Prep kit for Illumina (New England Bio-labs) following the protocol of the manufacturer. We pooled the final indexed libraries in equimolar ratios and sequenced them in a single lane of an Illumina XTen sequencing system (Illumina Inc.).

From the raw reads, we filtered out adaptors and low-quality reads in Trimmomatic v.0.33 (Bolger et al. 2014). We checked the quality of the remaining reads using FastQC (www.bioinformatics.babraham.ac.uk/projects/fastqc/) and performed *de novo* assembly in SPAdes 3.11 (Bankevich et al. 2012) with the k-mer of 75, 85, 95 and 105. A customised python script was employed (Jin et al. 2018) with its default parameters to apply BLAST and a built-in library to connect verified contigs into plastomes in SPAdes. We annotated the assembly of the resulting complete cp genomes using the Dual Organellar GenoMe Annotator (DOGMA) (Wyman et al. 2004) with *Glycyrrhizaglabra* L. [GenBank Accession #: NC_024038; Sabir et al. (2014)] as a reference (see Suppl. material 1 for details of the annotated cp genomes). Start and stop codons and intron/exon boundaries for protein-coding genes were checked manually. As our samples covered a vast phylogenetic range of clades within the IRLC legumes, the character of the cp genomes, such as genomic structure and gene order, varied dramatically (Sabir et al. 2014; Sveinsson and Cronk 2016). To avoid introducing potential error from the various genomic characters, we extracted 75 protein coding sequences (CDSs; as in Kang et al. 2018, except for *rps*12) from the annotated genomes and concatenated them with Geneious (Kearse et al. 2012) (see Table S2 in the supplementary file for details of the CDSs).

### Phylogenetic Analyses

We aligned the cp CDS sequences independently with MAFFT v.7 (Katoh and Standley 2013). The alignment was partitioned (Thode et al. 2020) and the best nucleotide substitution models for each CDS were inferred using PartitionFinder 2 (Lanfear et al. 2016) under the default settings (for the best models see Suppl. material 2). Accordingly, we carried out separate phylogenetic analyses using Bayesian Inference (BI; Rannala and Yang 1996; Mau et al. 1999) implemented in the programme MrBayes 3.2.5 (Ronquist and Huelsenbeck 2003; Ronquist et al. 2012) by applying default prior settings. Each BI was performed by applying two independent runs of the Markov Chain Monte Carlo (MCMC) for 10 million generations with sampling every 1,000 generations. We discarded the first 2,500 trees as burn-in and summarised the remaining posterior topologies as a maximum clade credibility (MCC) tree. The analysis stationarities were verified with Tracer v.1.6 (http://tree.bio.ed.ac.uk/software/tracer) by ensuring that all ESS values exceeded 200 and the convergence was confirmed between independent runs. In addition to BI, we also performed Maximum Likelihood (ML) analyses for cpCDSs, respectively, using IQ-TREE v.1.6 (Nguyen et al. 2015) with the following settings: rapid bootstrap analysis with 1,000 replicates, followed by a search for best-scoring ML tree starting with a random seed.

## Results

**Figure 2.**
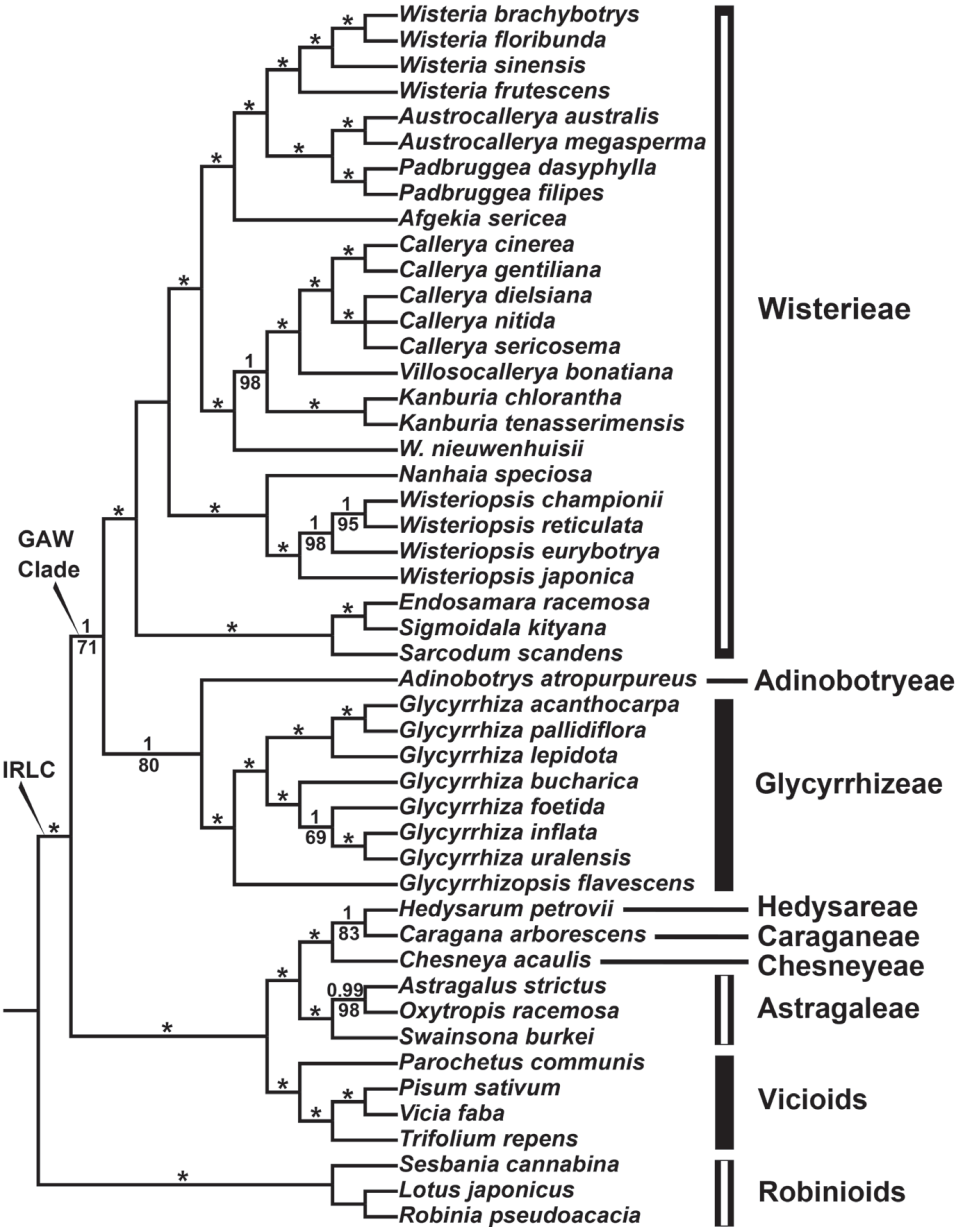
Bayesian maximum clade credibility tree of the GAW clade and related groups based on chloroplast CDSs. Bayesian posterior probabilities are given above branches, Maximum Likelihood bootstrap values below branches. Asterisks indicate PP = 1 and LBS = 100%. *W.nieuwenhuisii* indicates *Whitfordiodendronnieuwenhuisii*.

All the sequenced plastomes were successfully assembled into complete circular configurations. The sizes of the cp genomes ranged from 122,310 to 156,702 bp and the GC contents were between 33.7% and 35.9% (see Suppl. material 1 for details). The alignment length of concatenated cpCDSs for the analyses in MrBayes and IQ-TREE was 72,106 bp. Our ML results were congruent in topology with the corresponding BI trees, and the support values of the former (as “LBS” hereafter) were thus labelled on the corresponding branches of the latter (as “PP” hereafter). Phylogenetic reconstructions (Fig. 2) supported the monophyly of the IRLC (PP = 1, LBS = 100%), and the GAW clade (PP = 1, LBS = 71%) was sister to the rest of the IRLC taxa (PP = 1, LBS = 100%). Within the GAW clade, *Adinobotrys* and the well-supported Glycyrrhizeae (PP = 1, LBS = 100%) formed a clade (PP = 1, LBS = 80%), which was sister to tribe Wisterieae (PP = 1, LBS = 100%).

## Discussion

Within the GAW clade, some tropical/subtropical (rarely temperate) woody liana/tree species, formerly belonging to Millettieae s.l., clustered with the temperate herbaceous *Glycyrrhiza*-*Glycyrrhizopsis* clade (Compton et al. 2019; Duan et al. 2020, 2021; also see Fig. 2). Recently, Compton et al. (2019) and Duan et al. (2021) assigned the above-mentioned liana/tree group into two non-sister clades: genus *Adinobotrys* and tribe Wisterieae (14 genera), corroborated by our cpCDSs trees (Fig. 2). Herein, we propose a monogeneric tribe Adinobotryeae based on *Adinobotrys*, and resurrect the tribe Glycyrrhizeae corresponding to the *Glycyrrhiza*-*Glycyrrhizopsis* clade (see Taxonomic Treatment) for the following reasons:

**A.** Our phylogenomic analyses (Fig. 2) validated that *Adinobotrys* does not belong to Wisterieae; and the *Glycyrrhiza*-*Glycyrrhizopsis* clade is phylogenetically distant from both of the core groups of Galegeae [i.e. the *Erophaca*-Astragalean clade; see Lock and Schrire (2005)] and the type genus (i.e. *Galega* L.) (also see Wojciechowski et al. 2004; Duan et al. 2015, 2021; Compton et al. 2019). It is thus reasonable to divide the GAW clade into three independent groups to ensure monophyly of *Adinobotrys* and the *Glycyrrhiza*-*Glycyrrhizopsis* clade with respect to tribe Wisterieae.

**B.** Our recent analyses indicated that a chloroplast capture event may have occurred in the GAW clade, and the common ancestors of *Adinobotrys* and Wisterieae were hypothesised to be the putative paternal and maternal parents of the *Glycyrrhiza*-*Glycyrrhizopsis* clade, respectively (Duan et al. 2021). Since Wisterieae was already known as a well-defined tribe in the GAW clade, its counterpart groups (*Adinobotrys* and the *Glycyrrhiza*-*Glycyrrhizopsis* clade) should also be recognised at the tribal rank. Such an evolutionary event-linked taxonomy was also noted in previous studies (e.g. Degtjareva et al. 2012).

**C.** As far as morphological differentiation is concerned, Glycyrrhizeae, containing the widely distributed *Glycyrrhiza* and the Anatolian endemic *Glycyrrhizopsis* Boiss. (Meng 2005; Duan et al. 2020; see Fig. 1C–I), is easily distinguished from Wisterieae and *Adinobotrys* by its herbaceous habit and temperate distribution. *Adinobotrys*, which has often been treated as part of *Callerya* Endl. within Wisterieae (Polhill 1981; Schot 1994; Schrire 2005), has a unique evergreen tree habit in contrast to the predominantly liana habit of Wisterieae (Fig. 1A, B; also see Compton et al. 2019; Compton and Schrire 2020).

Despite the tribal revision above, taxonomic questions still remain. Since Glycyrrhizeae, and Astragaleae sensu Duan et al. (2021), corresponding to the aforementioned *Erophaca*-Astragalean clade, were removed from tribe Galegeae, the remaining genus *Galega* was found to be nested within the Vicioid clade (Steele and Wojciechowski 2003; Wojciechowski et al. 2004; Duan et al. 2021). Therefore, tribe Galegeae needs further taxonomic consideration before either being merged into a larger tribe corresponding to the Vicioid clade, or re-instated in its earlier, narrower sense, i.e. as the monogeneric tribe circumscribed by Hutchinson (1964). In addition, the recent work of Compton and Schrire (2020) expanded the genus *Adinobotrys* sensu Compton et al. (2019) to contain four species, although its infra-generic relationships need to be studied in more detail.

### Taxonomic treatment

#### Adinobotryeae

Taxon classificationPlantaeFabalesFabaceae

L.Duan, J.Compton & Schrire tr. nov.

9218AA37-A07B-5C6A-A448-0D460CE6C1F2

urn:lsid:ipni.org:names:77219547-1

Fig. 1A, B

##### Type.

*Adinobotrys* Dunn, Bull. Misc. Inform. 1911: 194. 1911.

##### Diagnosis and note.

Compared to the tribe Wisterieae, the monogeneric Adinobotryeae comprises four species of evergreen trees (vs. lianas in tribe Wisterieae). The species are: *A.atropurpureus* (Wall.) Dunn, *A.katinganensis* (Adema) J.Compton & Schrire, *A.sarawakensis* (Adema) J.Compton & Schrire and *A.vastus* (Kosterm.) J.Compton & Schrire. See the detailed description of *Adinobotrys* in Compton et al. (2019: 49) and a diagnostic key to the species and full list of synonymy in Compton and Schrire (2020).

##### Description.

Evergreen trees, up to 20 m in height. Stipules triangular, persistent. Stipels absent. Leaves 5–9 (–11) foliolate; rachis 11–33 cm long; leaflets 5–21 × 2–11 cm, coriaceous, ovate, elliptic to obovate, glabrous, apex acuminate, base obtuse to cordate. Inflorescence a terminal panicle 10–40 cm long; bracts 2–4 mm long, ovate; peduncle sparsely hairy to tomentose. Flowers 14–20 mm long; bracteoles at base of calyx tube, persistent, ovate; pedicels densely pubescent. Calyx narrowly campanulate, oblique, green, puberulent, five lobes acute to obtuse. Standard 11–20 × 13–20 mm, broadly ovate, apex acute, outer surface glabrous, inner surface pink to reddish-purple, rarely white, nectar guide yellow, with callosities; wings 12–19 × 5–8 mm, glabrous; keel ± equal to wings in length, glabrous, apex acute to rounded. Stamens diadelphous, vexillary filament free from other nine, all curved upwards at apex. Ovary hairy; style glabrous. Pods 7–25 × 3–6 cm, dehiscent, inflated or compressed, irregularly ovate to oblong or narrowly elliptic, surface glabrous, rugose, subseptate. Seeds 1–4 per pod, ovoid to oblong or flattened-orbicular, 15–38 mm long.

##### Distribution and habitat.

Evergreen forests in Brunei, Cambodia, India, Indonesia (Java, Kalimantan and Sumatra), Laos, Malaysia (Peninsula and Sabah), Myanmar, Thailand and Vietnam, below 1200 m in altitude.

#### Glycyrrhizeae

Taxon classificationPlantaeFabalesFabaceae

Rydb., Fl. Rocky Mts. 454. 1917.

3B06EFA7-AF35-54B7-AC68-410356ADEC27

Fig. 1C–I

≡ Glycyrrhizinae Rydb., N. Amer. Fl. 24(3): 156. 1923.

##### Type.

*Glycyrrhiza* L., Sp. Pl. 2: 741. 1753.

##### Distribution and habitat.

Mediterranean, warm temperate and continental temperate grasslands, shrublands, deserts and forest edges in the Old World [from the western Mediterranean region (Iberian peninsula in Europe and Algeria in North Africa), through the Russian Far East, Mongolia and northern China (plus Sichuan and Yunnan of south-western China) to Australia (*Glycyrrhizaacanthocarpa* J.M.Black), including the States of Queensland, New South Wales, Victoria, South Australia and Western Australia] and the New World [in western temperate Canada and the United States (*G.lepidota* Pursh) and in the temperate region of Argentina and Chile (around 40°S; *G.astragalina* Gillies)].

##### Note.

The tribe includes two genera, *Glycyrrhiza* and *Glycyrrhizopsis*, with the latter confined in S. Anatolia. The root of *Glycyrrhiza* is widely used as medicine and in the food industry (see Duan et al. 2020).

## Supplementary Material

XML Treatment for Adinobotryeae

XML Treatment for Glycyrrhizeae

## Supplementary materials

Supplementary material 1 Voucher information, characters of chloroplast (cp) genomes and GenBank BioSample accession of genome skimming raw reads in this study Data type XLSX. file Explanation note Voucher information, characters of chloroplast (cp) genomes and GenBank BioSample accession of genome skimming raw reads in this study. https://binary.pensoft.net/file/583808This dataset is made available under the Open Database License (http://opendatacommons.org/licenses/odbl/1.0/). The Open Database License (ODbL) is a license agreement intended to allow users to freely share, modify, and use this Dataset while maintaining this same freedom for others, provided that the original source and author(s) are credited.Lei Duan, Li-Na Han, Yotsawate Sirichamorn, Jun Wen, James A. Compton, Shuang-Wen Deng, Emine Arslan, Kuddisi Ertuğrul, Brian Schrire, Hong-Feng Chen

Supplementary material 2 Chloroplast protein coding sequences (cpCDSs) and their corresponding substitution models generated from PartitionFinder 2 Data type XLSX. file Explanation note Chloroplast protein coding sequences (cpCDSs) and their corresponding substitution models generated from PartitionFinder 2. https://binary.pensoft.net/file/583809This dataset is made available under the Open Database License (http://opendatacommons.org/licenses/odbl/1.0/). The Open Database License (ODbL) is a license agreement intended to allow users to freely share, modify, and use this Dataset while maintaining this same freedom for others, provided that the original source and author(s) are credited.Lei Duan, Li-Na Han, Yotsawate Sirichamorn, Jun Wen, James A. Compton, Shuang-Wen Deng, Emine Arslan, Kuddisi Ertuğrul, Brian Schrire, Hong-Feng Chen
